# Comparative study on the modulation of incretin and insulin homeostasis by butyrate in chicken and rabbit

**DOI:** 10.1371/journal.pone.0205512

**Published:** 2018-10-11

**Authors:** Gábor Mátis, Anna Kulcsár, Máté Mackei, Janka Petrilla, Zsuzsanna Neogrády

**Affiliations:** Division of Biochemistry, Department of Physiology and Biochemistry, University of Veterinary Medicine, István utca 2, Budapest, Hungary; University of Michigan, UNITED STATES

## Abstract

The pancreatic secretion of insulin, a key endocrine regulator of metabolism and growth, can be greatly influenced by the gut-derived incretin hormones, namely by GIP (Glucose-dependent Insulinotropic Peptide) and GLP-1 (Glucagon-like Peptide 1). As insulin is a major stimulator of growth, affecting its producion may be of special importance in food-producing livestock. The aim of the present study was to investigate novel ways of modulating incretin and insulin homeostasis in chickens and rabbits by nutrition, e.g. by oral butyrate application, also studying the mechanisms of incretin action in both species as a comparative approach. Acute oral butyrate challenge significantly decreased plasma GIP levels by approx. 40% in both species: significant interactions of butyrate exposure and incubation time were found in both chickens (*P* = 0.038 and *P* = 0.034 at 30 and 60 min following butyrate ingestion [1.25 g/kg BW], respectively) and rabbits (*P* = 0.036 and *P* = 0.039 at 30 and 60 min after butyrate ingestion [0.25 g/kg BW], respectively), while plasma GLP-1, insulin and glucose concentrations remained unaffected by butyrate in both species over time. These results are in contrast to butyrate’s stimulating effect on both incretin and insulin secretion in mice, indicating specific, species-dependent differences even among mammalian species. Further, based on the analyzed correlations between the measured endocrine parameters (regardless of the butyrate exposure), it can be assumed that incretins may regulate pancreatic insulin release in rabbits on a partly different way compared to mice, humans and chickens.

## Introduction

Insulin plays pivotal role in the regulation of carbohydrate and lipid metabolism, while it is also considered as an indispensable stimulator of growth by increasing protein synthesis and affecting the expression of several growth-related genes [1]. In both mammals and birds, pancreatic insulin release is highly controlled by the gut-derived incretin hormones, e.g. by GIP (Glucose-dependent Insulinotropic Peptide) and GLP-1 (Glucagon-like Peptide 1) as key members of the enteroinsular axis [2]. GIP is produced by the K cells of the small intestines and stimulates the insulin secretion of pancreatic β cells together with GLP-1, the latter released from L cells, expressed in small and large intestines as well [3,4]. Further, GLP-1 can enhance insulin synthesis by increasing insulin gene expression and also influences differentiation and proliferation of β cells [5]. The way of incretin action is mostly known from model studies with rodents, while only limited data are available with regard on domestic animal species. Since the carbohydrate metabolism of birds highly differs from that of mammals, featuring decreased plasma insulin level and tissue insulin sensitivity combined with increased plasma glucose concentration [6], some differences do exist concerning the regulatory role of incretins between mammals and birds. For instance, GLP-1 elicits its insulinotropic action in chicken more likely by influencing the somatostatin production of pancreatic δ cells rather than by direct β cell stimulation [7].

Based on the anabolic properties of insulin, influencing its secretion by nutrition via the enteral incretin production may provide new ways to improve animal production. The short chain fatty acid (SCFA) butyrate, which is a commonly applied growth promoting feed additive, can stimulate incretin and insulin secretion [8] as well as insulin sensitivity in mice [9]. In contrast, the chronic oral uptake of SCFAs could decrease plasma GIP and GLP-1 levels in the same species [10]. As butyrate is also known to influence insulin signaling of chickens in a tissue-dependent manner by selectively up-regulating insulin receptor β subunit (IRβ) in skeletal muscle, this molecule can be considered as a potent effector of insulin homeostasis in birds as well [11]. In chicken, there are only limited data available with regard on influencing the incretin homeostasis, but a recent study revealed that *in ovo* application of novel synbiotics (composed of *Lactobacillus* spp. and oligosaccharides) significantly decreased intestinal GIP and GLP-1 production in 6-week-old broilers [12].

In association with the suggested species-dependent differences, investigating the molecular mechanisms of insulin and incretin homeostasis in various species can have great importance from comparative physiological approach. Therefore, the main goal of this study was to assess the effects of acute oral butyrate treatment on incretin and insulin secretion in chicken and rabbit. These two species were selected as being potential targets of butyrate administration as a feed additive and also serving as models for birds and mammals. Studying novel possibilities for nutritional modulation of the incretin homeostasis may also provide new data regarding the regulatory role of incretins on pancreatic insulin release in different species, and may highlight new data about the mechanisms of incretin action.

## Materials and methods

### Ethics statement

All animal procedures reported herein were conducted in accordance with international and national laws as well as with institutional guidelines. Husbandry and experimental procedures were approved by the Government Office of Pest County, Food Chain Safety, Plant Protection and Soil Conservation Directorate, Budapest, Hungary (number of permission: PEI/001/1430-4/2015).

### Animals, treatments and samplings

#### Chicken experiment

Newly hatched male broiler chicks (n = 21 totally) of the Ross 308 strain, obtained from a commercial hatchery (Bábolna Tetra Ltd., Uraiújfalu, Hungary), were randomized into 3 experimental groups and were housed in floor pens on chopped wheat straw; housing and climatic circumstances were set according to the Ross technology over the entire trial [13]. Chickens were allocated to experimental groups randomly, but it was controlled that no significant differences could be observed in body weight between any groups. Feed and water were provided *ad libitum*; the applied diet was formulated based on the requirements of the breed [13]. Calculated nutrient composition of the diet is shown in Table 1.

**Table 1:** Calculated nutrient composition of the diet of chickens.

Item	
Calculated nutrient composition (fresh basis)	
Crude protein, g/kg	212.2
Ether extract, g/kg	29.4
Crude fiber, g/kg	25.3
Ash, g/kg	65.9
AME, MJ/kg	11.9
Lysine, g/kg	11.9
Methionine, g/kg	4.9
Methionine + Cysteine, g/kg	8.6
Calcium, g/kg	11.6
Available phosphorus, g/kg	4.5

On day 24, animals were treated following overnight feed deprivation with a single intra-ingluvial sodium butyrate bolus in two different doses, administered by a tube. A randomly selected group of chickens received a dose of 0.25 g sodium butyrate/kg BW (referring to the average dose used in poultry nutrition; applied in a solution of 0.1 g/mL, 2.5 mL/kg BW; n = 7), while a higher dose of sodium butyrate was given to another group (1.25 g/kg BW, ingested in a solution of 0.5 g/mL, administered as 2.5 mL/kg BW; n = 7) to test the dose-dependency of butyrate’s action. Physiological saline solution was applied for seven chicks under the same conditions as a control group (2.5 mL/kg BW; n = 7). Butyrate administration in a daily oral bolus (following overnight feed deprivation) is an already approved treatment model, providing a fast, short-term butyrate exposure and being suitable for investigations on butyrate’s *in vivo* metabolic actions [14].

Blood samples were drawn from the brachial vein of chickens into heparinized tubes at the following time points: prior to the butyrate exposure (0 min); 10, 30 and 60 min following the bolus treatment. After immediate centrifugation (1000 x g, 10 min), plasma samples were stored at -80°C until further processing.

#### Rabbit experiment

Six-week-old male rabbits (n = 24 totally) of the Pannonian Giant breed (obtained from Anas Ltd., Nagyhajmás, Hungary) were randomized into 3 experimental groups and were housed in floor pens under controlled environmental conditions according to the requirements of the breed. Similarly to chickens, rabbits were also allocated to groups randomly, but controlled for body weight. Animals had free access to the supplied pelleted diet (Table 2), hay and drinking water.

**Table 2:** Calculated nutrient composition of the diet of rabbits.

Item	
Calculated nutrient composition (fresh basis)	
Crude protein, g/kg	172.0
Ether extract, g/kg	29.5
Crude fiber, g/kg	154.9
Ash, g/kg	104.5
DE, MJ/kg	10.6
Lysine, g/kg	7.6
Methionine, g/kg	3.9
Methionine + Cysteine, g/kg	7.2
Calcium, g/kg	9.6
Available phosphorus, g/kg	4.6

At the age of 7 weeks, after overnight fasting, rabbits were exposed to a single sodium butyrate bolus given by a gastric tube with the same dosage as administered in the chicken experiment (0.25 g/kg BW and 1.25 g/kg BW; n = 8/group), while control animals were treated with physiological saline solution (2.5 mL/kg BW; n = 8).

Blood samplings were conducted similarly to the chicken study; samples were taken from the marginal ear vein before butyrate ingestion (0 min) and 10, 30 and 60 min following the bolus exposure. Heparinized plasma samples were stored at -80°C until analysis.

### Measurement of plasma GIP, GLP-1, insulin and glucose concentrations

The GIP, GLP-1 and insulin concentrations of blood plasma samples were assessed by chicken- and rabbit-specific sandwich ELISA kits, purchased from MyBioSource (San Diego, CA, USA; Cat. No.: chicken insulin: MBS701713, chicken GIP: MBS261884, chicken GLP-1: MBS260694; rabbit insulin: MBS704952, rabbit GIP: MBS2600212, rabbit GLP-1: MBS2512151) with intra- and inter-assay variations below 15%, according to the manufacturer’s instructions (calculated actual values were all in this range, confirming the accuracy of the analyses). Blood glucose concentrations were measured by the colorimetric Fluitest Glucose Assay (Analyticon, Lichtenfels, Germany).

### Statistics

All values are expressed as means ± SEM. Statistical analysis of data was performed with the R 2.14.0 software, by a randomized linear mixed model. The possible interactions between treatment and incubation time were assessed by the applied model; significant interactions indicated that the appropriate butyrate treatment significantly influenced the time course of the measured parameter at a given time point compared to that of controls. Correlations between the measured parameters were analyzed by Pearson’s correlation test; the obtained results were also confirmed by randomized linear mixed models. Level of significance was set at P < 0.05.

## Results

Considering the effect of the incubation time on the measured endocrine parameters, GIP and GLP-1 were not influenced by time, while plasma insulin level significantly decreased with time in chickens and rabbits as well (P = 0.002 and P = 0.016, respectively).

**Fig 1:**
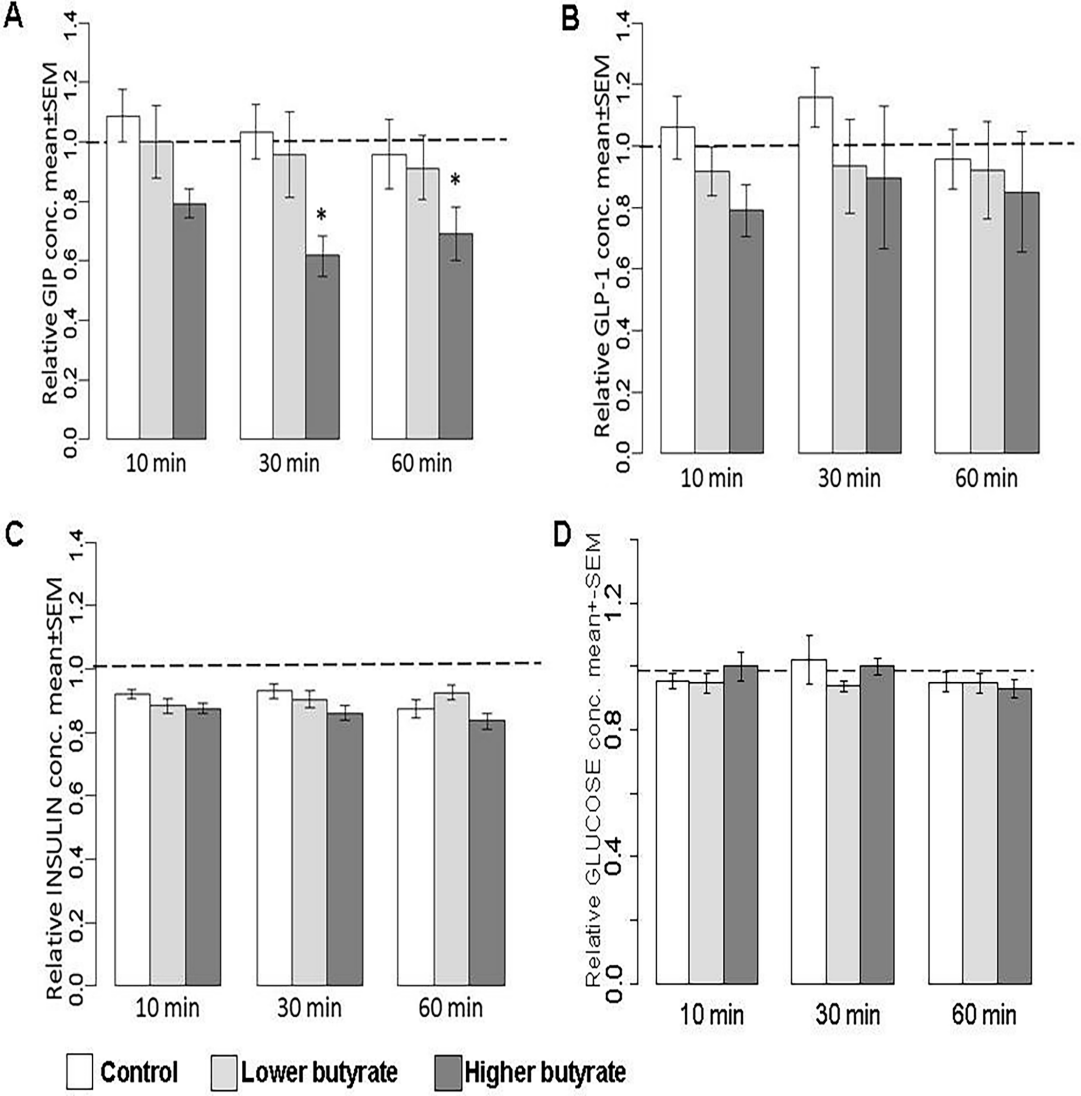
**Relative concentrations of A. GIP, B. GLP-1, C. insulin and D. glucose in the blood plasma of chickens.** Sodium butyrate was applied in the doses of 0.25 g/kg BW (referring to “Lower butyrate”) and 1.25 g/kg BW (indicated by “Higher butyrate”). Relative hormone concentrations were calculated by considering the baseline value of each animal at 0 min as 1. Results are expressed as mean ± SEM. Statistical analysis of data was performed by a randomized linear mixed model, asterisks indicate statistical significance compared to the 0 min values of the appropriate group (interaction between time and treatment), *P < 0.05.

**Fig 2:**
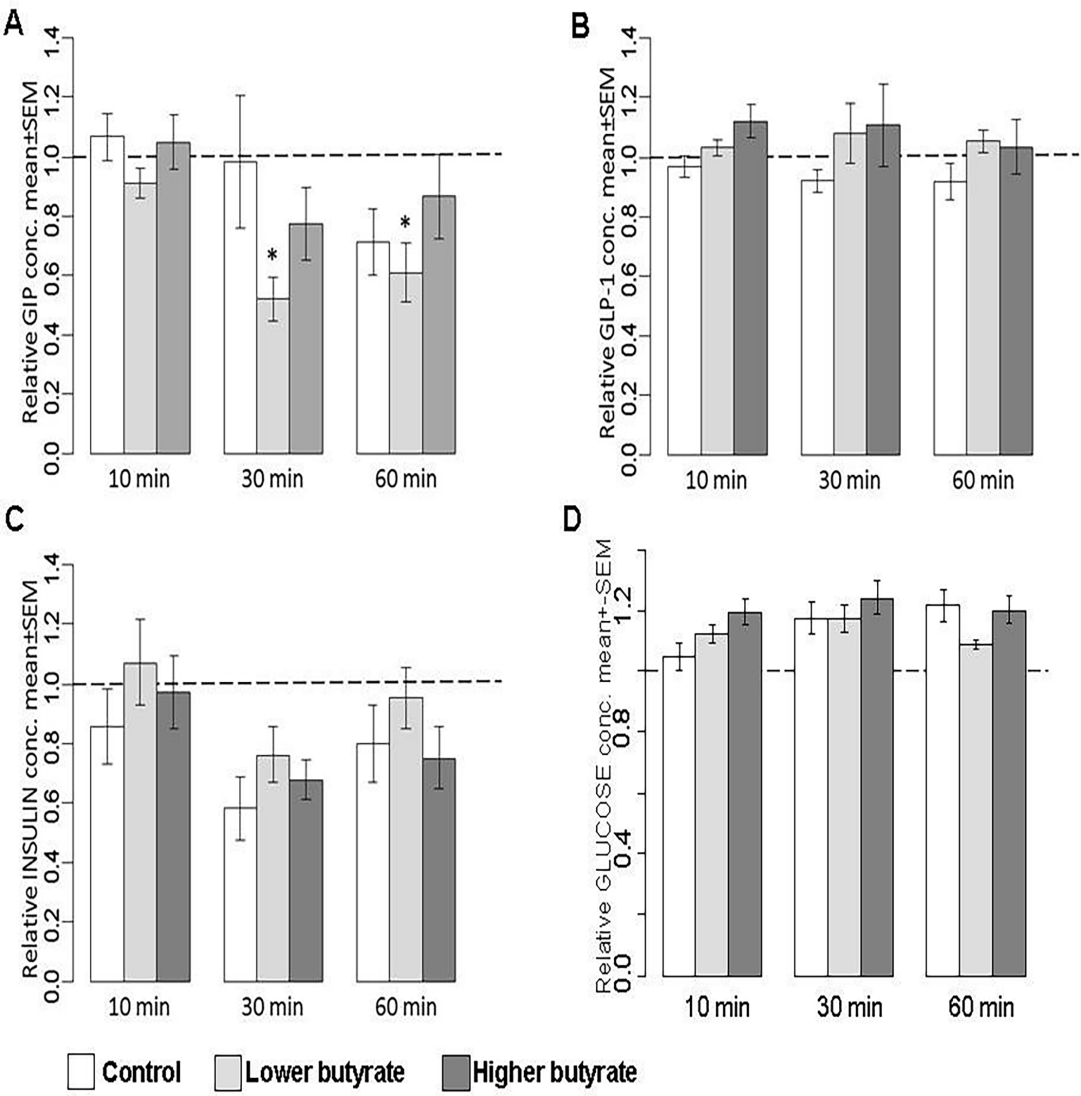
**Relative concentrations of A. GIP, B. GLP-1, C. insulin and D. glucose in the blood plasma of rabbits.** Sodium butyrate was applied in the doses of 0.25 g/kg BW (referring to “Lower butyrate”) and 1.25 g/kg BW (indicated by “Higher butyrate”). Relative hormone concentrations were calculated by considering the baseline value of each animal at 0 min as 1. Results are expressed as mean ± SEM. Statistical analysis of data was performed by a randomized linear mixed model, asterisks indicate statistical significance compared to the 0 min values of the appropriate group (interaction between time and treatment), *P < 0.05.

Concentration of GIP in blood plasma of chickens was remarkably affected by butyrate exposure: significant interactions were detected between the higher dose (1.25 g/kg BW) of butyrate and incubation time (P = 0.038 and P = 0.034 for 30 and 60 min, respectively). These interactions are reflected by the butyrate-associated decrease of plasma GIP levels at 30 and 60 min with approx. 40% compared to 0 min values (Fig 1A). Similar significant interactions (P = 0.036 and P = 0.039 for 30 and 60 min, respectively) could be observed in rabbits, where plasma GIP concentrations were lowered 30 and 60 min after ingestion of the lower dose of butyrate (0.25 g/kg BW) with approx. 45% when compared to the initial baseline values (Fig 2A).

Regarding the GLP-1 and insulin concentrations, no significant interactions were observed between butyrate exposure and incubation time, so, according to our results, butyrate had no significant effects on GLP-1 and insulin levels either in chickens or in rabbits. Results obtained by GLP-1 measurements of chickens and rabbits are presented in Figs 1B and 2B, respectively; relative values of plasma insulin concentrations can be seen in Figs 1C and 2C.

Blood glucose concentrations of chickens were not altered by time, and there were no significant interactions between any time points and treatments (Fig 1D). In rabbits, blood glucose concentrations significantly increased with time (P = 0.002), but they were not affected by the butyrate exposure (Fig 2D). Time zero concentrations of all hormones and those of glucose are indicated in Table 3 for chickens and in Table 4 for rabbits.

**Table 3:** Concentration of GIP, GLP-1, insulin and glucose in the blood plasma of chickens at 0 min.

	GIP (pg/mL)	GLP-1 (pg/mL)	Insulin (μIU/mL)	Glucose (mmol/L)
Control	46.56 ± 6.44	9.62 ± 1.63	10.05 ± 0.15	11.49 ± 0.35
Butyrate, 0.25 g/kg BW	35.41 ± 2.24	9.20 ± 0.90	9.70 ± 0.16	10.54 ± 0.28
Butyrate, 1.25 g/kg BW	36.21 ± 3.79	7.05 ± 1.29	9.56 ± 0.28	11.20 ± 0.51

Results are expressed as mean ± SEM.

**Table 4:** Concentration of GIP, GLP-1, insulin and glucose in the blood plasma of rabbits at 0 min.

	GIP (pg/mL)	GLP-1 (pg/mL)	Insulin (pg/mL)	Glucose (mmol/L)
Control	58.85 ± 12.69	96.65 ± 10.07	237.86 ± 59.18	5.53 ± 0.16
Butyrate, 0.25 g/kg BW	75.50 ± 11.30	86.78 ± 4.29	117.15 ± 17.32	6.01 ± 0.18*
Butyrate, 1.25 g/kg BW	55.86 ± 10.08	99.76 ± 8.31	126.62 ± 7.39	5.80 ± 0.12

Results are expressed as mean ± SEM.

*P<0.05.

**Fig 3:**
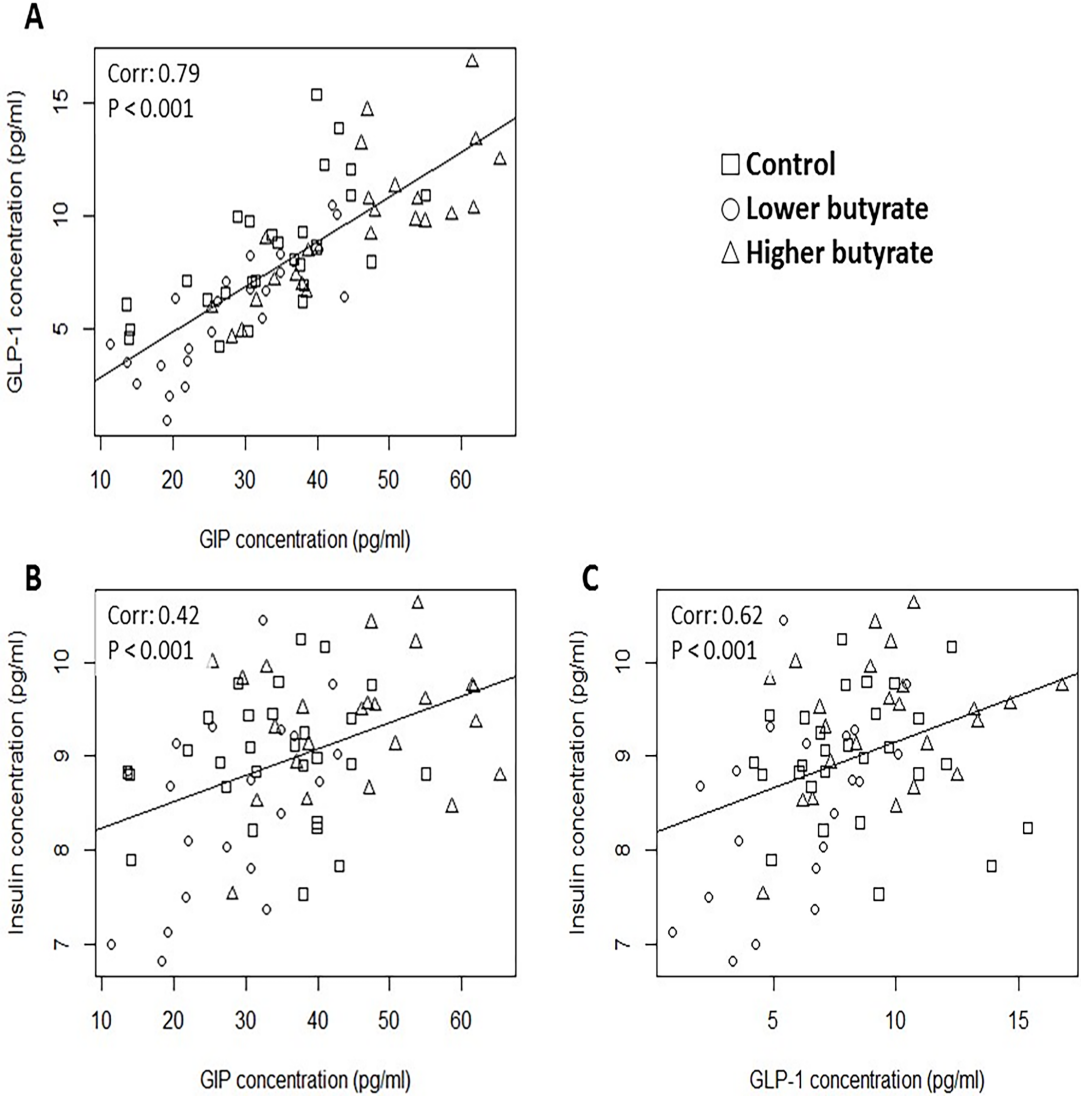
**Correlation between A. GIP and GLP-1, B. GIP and insulin, C. GLP-1 and insulin concentrations in the blood plasma of chickens.** Each dot refers to an individual animal according to its blood plasma hormone concentrations, indicated on the axes. Correlations were statistically analyzed by Pearson’s correlation test. The obtained correlation coefficients and *P* values were as follows: A. Coefficient: 0.79, P < 0.001; B. Coefficient: 0.42, P < 0.001; C. Coefficient: 0.62, P < 0.001.

**Fig 4:**
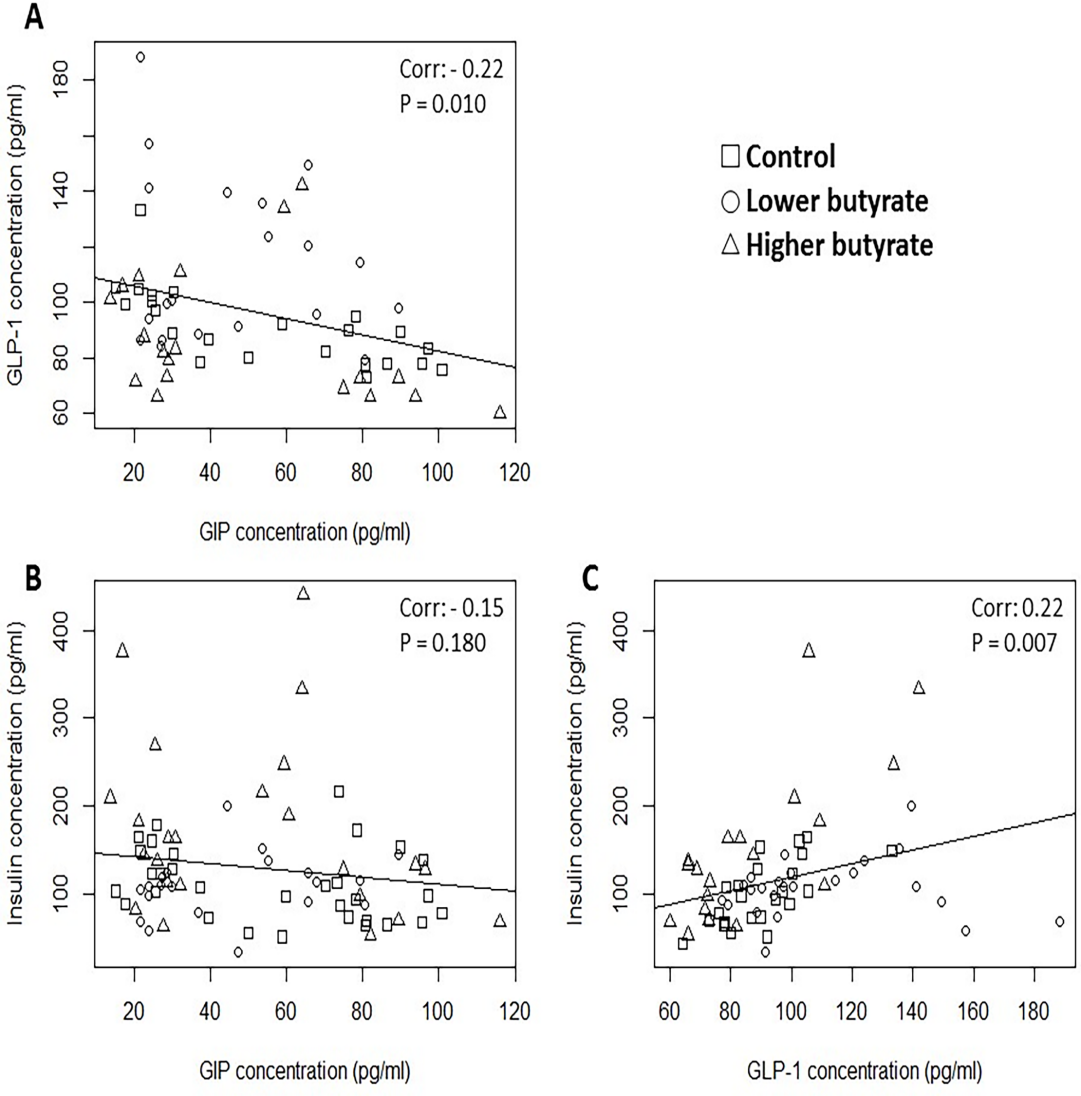
**Correlation between A. GIP and GLP-1, B. GIP and insulin, C. GLP-1 and insulin concentrations in the blood plasma of rabbits.** Each data point refers to an individual animal according to its blood plasma hormone concentrations indicated on the axes. Squares refer to controls, circles to animals receiving the lower dose (0.25 g/kg BW) of butyrate and triangles to animals exposed to the higher dose (1.25 g/kg BW) of butyrate. Correlations were statistically analyzed by Pearson’s correlation test. The obtained correlation coefficients and P values were as follows: A. Coefficient: -0.22, P = 0.010; B. Coefficient: -0.15, P = 0.180; C. Coefficient: 0.22, P = 0.007.

Concerning the correlations between the measured endocrine parameters in chickens (regardless of the butyrate exposure), highly significant (P < 0.001) positive correlations were found between plasma GIP and GLP-1 values (Fig 3A), GIP and insulin levels (Fig 3B) and GLP-1 and insulin concentrations (Fig 3C). In rabbits, a significant negative correlation was observed between plasma GIP and GLP-1 levels (Fig 4A, P = 0.010), while there was no significant correlation between GIP and insulin values (Fig 4B, P = 0.180). However, plasma GLP-1 and insulin concentrations positively correlated in a significant manner (Fig 4C, P = 0.007) in rabbits as well.

## Discussion

It has been described that orally applied butyrate as a potent effector of carbohydrate and lipid metabolism can increase both insulin secretion and insulin sensitivity in mice [9], mediated mostly by the stimulation of intestinal incretin secretion [8]. Influencing both the pancreatic production and the cellular signaling of insulin may greatly improve its efficacy in the regulation of metabolism and growth. However, butyrate significantly decreased the expression of key insulin signaling proteins in the liver of chickens, but exclusively up-regulated IRβ in skeletal muscle [11], revealing the species- and tissue-dependency of butyrate’s action on insulin homeostasis. Based on these findings, the aim of the present study was to provide more data concerning the effects of orally applied butyrate on insulin secretion and its major mediators, the incretins in chickens and rabbits as target species of butyrate application. In addition, comparing the butyrate-associated changes in the endocrine regulatory mechanisms may help to get a better insight towards the species-related differences of incretin action.

Our results regarding the interaction of butyrate exposure and incubation time showed that orally applied butyrate affected plasma GIP concentrations in both chickens and rabbits (Figs 1A and 2A, respectively), but no significant effects could be detected with regard on the GLP-1 and insulin levels (Fig 1B and 1C, Fig 2B and 2C). The direction, extent and time course of the butyrate-associated changes seemed to be similar in both examined species as an approx. 40% decrease in plasma GIP concentration at the sampling points of 30 and 60 min following butyrate exposure. Even these intense alterations of GIP levels were not realized in significant changes of the plasma insulin concentration, confirming that GIP plays only a partial role in the complex neuroendocrine regulation of insulin release.

The observed butyrate-associated reductions in plasma GIP levels are in contrast with the findings of previous studies in mice, where similarly applied butyrate significantly increased the plasma concentrations of both incretins and insulin [8]. Notwithstanding that further studies are needed to explain the observed differences between mice and the examined species of our trial, they might be in association with some species-related differences in carbohydrate metabolism. There are clear and remarkable differences between the insulin homeostasis of mammals and birds, but in this case various mammalian species (mice and rabbits) responded to acute butyrate exposure in a different manner, while chickens and rabbits on a similar way. However, it should be also taken into account that 3-month-old mice (considered as young adults) were used in the trial of Lin et al. [8], while both rabbits and chickens of the present study were in the phase of intensive growth.

According to the species-specific dietary requirements, rabbit diets always contain higher amount of dietary fibers than those of mice or chickens, providing more substrate for the microbial SCFA production in the large intestines. Therefore, a relevant amount of butyrate is produced in the caecum of rabbits [15], possibly making the tissues more adapted and being less sensitive to butyrate compared to mice and chickens. Chicken and rabbit were chosen in our present study, because they are both target species of oral butyrate supplementation as a feed additive [16, 17]; further, they can also serve as avian and mammalian models in metabolic studies. Applying rabbits instead of the most common rodents as models provides more possibilities for inter-species comparisons (newly gained data of rabbits can be compared to those of previous rat and mouse studies and not only to the present results of chickens). However, the intense caecal bacterial digestion in rabbits should be also addressed as a limitation when considering the inter-species differences and applying rabbit as a model species.

The effects of SCFAs on incretin homeostasis can be also diverse even in the same species. Continuous, chronic dietary exposure to SCFAs significantly decreased plasma incretin levels in mice [10], and feeding a fiber-rich diet (stimulating intestinal SCFA production) could also diminish plasma GIP concentration in rats [18]. Based on these data, it should be addressed that the intestinal expression of SCFA receptors (such as FFAR2 and FFAR3), the way of SCFA application (acute or chronic challenge, stimulating intestinal fermentation) may also contribute to the observed differences of incretin response [10, 19]. It was also considered that the age might play a role in the changes of incretin and insulin secretion. Therefore, both chickens and rabbits were investigated in our study in the phase of intensive growth to get a reliable comparability.

When examining the effects of oral butyrate exposure on plasma GIP concentrations in chickens and rabbits, it can be seen that the higher butyrate dose (1.25 g/kg BW) was required to cause significant alterations in chickens, while only the lower butyrate dose (0.25 g/kg BW) was capable to similarly reduce GIP levels in rabbits. This difference might be explained by the mentioned intensive intestinal butyrate production of rabbits [15]. The high amount of endogenously produced gut-derived butyrate together with the orally ingested one results in much more elevated plasma and tissue butyrate concentrations in rabbits than in chickens. It is also known that especially high concentrations of butyrate may have no or adverse effects on several metabolic processes compared to lower doses, already stated in numerous *in vitro* and *in vivo* studies. For instance, applying a daily oral bolus of butyrate for 5 days affected the acetylation rate of hepatic histones in chickens in a dose-dependent manner [14]. The acetylation of histone H3 was stimulated only by the higher dose (1.25 g/kg BW) of butyrate, while exclusively the lower dose (0.25 g/kg BW) could increase the acetylation state of histone H4 [14]. Similarly, butyrate applied orally in the dose of 0.25 g/kg BW significantly ameliorated the enzyme inducing action of phenobarbital on hepatic cytochrome P450 enzymes in chicken, but this effect was absent when given in the dose of 1.25 g/kg BW [20]. The lack of butyrate’s action at higher concentrations was also found in certain bacterial strains, such as in *Rhodopseum faecalis* RLD-53, where butyrate supplied at lower levels increased hydrogen production, but not at elevated concentrations [21]. Dose-associated alterations of butyrate’s action may be in connection with its dose-dependent epigenetic effects on histone deacetylase and histone acetyltransferase enzymes influencing histone acetylation, playing central role in the mediation of butyrate’s major actions [22].

The allocation of the animals to experimental groups was carried out randomly, but controlled for the body weight ensuring no significant differences between any groups. However, the baseline (0 min) plasma concentration of the measured parameters was not taken into account for the allocation, but no significant differences were observed between any groups at 0 min concerning all measures except one. Plasma glucose concentration of rabbits exposed to the lower dose of butyrate was significantly higher than that of controls at 0 min (P = 0.011, 6.01 ± 0.18 mmol/l vs. 5.53 ± 0.16 mmol/l), but this difference cannot be considered as a relevant one and suggested to play no role in the observed endocrine alterations. The further observable numerical differences (e.g. GIP and insulin in rabbits) did not reach the level of statistical significance. Plasma glucose concentrations were increased with time in rabbits, independently of the butyrate exposure. This can be in association with the stress-sensitivity of rabbits, despite of the minimized pain and stress during repeated blood samplings.

Concerning the correlations between the measured endocrine parameters, some major differences were found between chickens and rabbits. GLP-1 and insulin were positively correlated in both chickens and rabbits (Figs 3C and 4C, respectively), justifying the expected stimulatory role of GLP-1 in pancreatic insulin secretion. However, GIP and insulin showed a positive correlation in chickens (Fig 3B), but this correlation was completely missing in rabbits (Fig 4B). In chickens, there was a strong positive correlation between GIP and GLP-1 (Fig 3A), which was lacking in rabbits and was replaced by a negative correlation (Fig 4A).

These findings highlight that there might be species-specific differences in the action of incretins on pancreatic insulin secretion, being an important point from comparative physiological approach. In chickens, the detected positive correlations confirm the presumed synergistic inducing action of both GIP and GLP-1 on β cell insulin release. However, it can be assumed that GIP may not play the same major insulinotropic role in rabbit, differing from chicken, mouse and human [23, 24].

Concluding the present results, it has been justified that butyrate has a significant role in influencing insulin homeostasis in both chickens and rabbits, which is suggested to be partly mediated by incretins. It can be stated that butyrate may have different effects on incretin and insulin secretion in various species, presenting differences even among mammalian species. In contrast to butyrate’s stimulatory action on incretin production in mice, acute oral butyrate exposure significantly decreased plasma GIP levels in both chickens and rabbits, but had no effect on GLP-1, insulin and glucose concentrations of blood plasma. Therefore, it is suggested that the nutritional modulation of insulin secretion should be specifically investigated in each target species, and results from model studies may be extrapolated to other species only with strong limitations. In addition, it can be assumed based on the analyzed correlations that incretins may regulate pancreatic insulin release on a partly different way in rabbits compared to other examined mammals and chickens: the major stimulatory action of GIP on insulin secretion may be questionable in rabbits according to the lacking correlation of GIP and insulin and the negative correlation between the two incretin hormones.

## Supporting information

S1 Table Concentration of glucose, insulin, GLP-1 and GIP in the blood plasma of chickens at different time points (raw data). (XLSX)

S2 Table Concentration of glucose, insulin, GLP-1 and GIP in the blood plasma of rabbits at different time points (raw data). (XLSX)
